# Electrocoagulation removal of Pb, Cd, and Cu ions from wastewater using a new configuration of electrodes

**DOI:** 10.1016/j.mex.2022.101951

**Published:** 2022-12-02

**Authors:** Forat Yasir AlJaberi, Zahraa Alaa Hawaas

**Affiliations:** Chemical Engineering Department, College of Engineering, Al- Muthanna University, Al- Muthanna, Iraq

**Keywords:** Toxic metals, Electrocoagulation technology, Bipolar, RSM-BBD, Analysis and optimization, Electrocoagulation removal of Pb, Cd, and Cu ions from wastewater using a new configuration of electrodes

## Abstract

A new configuration of aluminum electrodes has been performed in an electrocoagulation reactor (ECR) to remove toxic metals from synthetic wastewater. The ECR contains four concentric-cubic electrodes that were connected to the DC power supply with a bipolar mode. The ability of this reactor to eliminate 200 ppm Pb, 200 ppm Cd and 200 ppm Cu from wastewater was investigated under the effect of pH (4–10), applied current (0.2–2.6 A), and the reaction time of (4–60 min). Two grams of NaCl were added to each experiment to enhance the electrical conductivity and minimize the passivation of cathode surfaces. The experiments, analysis, and optimization were conducted using response surface methodology type Box-Behnken design (RSM-BBD) and the Minitab-statistical software program. The highest elimination of heavy metals was: Pb-99.73%, Cd-98.54%, and Cu-98.92% at pH 10, 1.4 A of the applied current, and 60 min of the reaction time. The total real consumption of anodes under these conditions was 0.55 g, and the energy consumption was 12.71 kWh/m3. All reactions of metal removal that occurred in the present EC reactor obey the kinetic of a first-order reaction. Thermodynamics parameters of present electrocoagulation removal of heavy metals indicate an endothermic, spontaneous nature, and random irregularity at the liquid-solid interaction. The highest values of removal efficiencies and the considerably lowest values of energy and electrode consumption proved that the electrocoagulation technology applies in wastewater treatment containing toxic metals.

• The anode electrodes were perforated to decrease the amount of electrode consumption, while the cathode electrodes were not perforated.
• The new EC reactor eliminated Pb-99.73%, Cd-98.54%, and Cu-98.92% of 200 mg/l of each metal at pH 10, applied current of 1.4 A, and reaction time of 60 min. Moreover, the consumption of energy and electrodes was significantly low.
• The performance indicator (R^2^) of the studied responses was higher than 0.95.

## Graphical abstract

**Figure:**
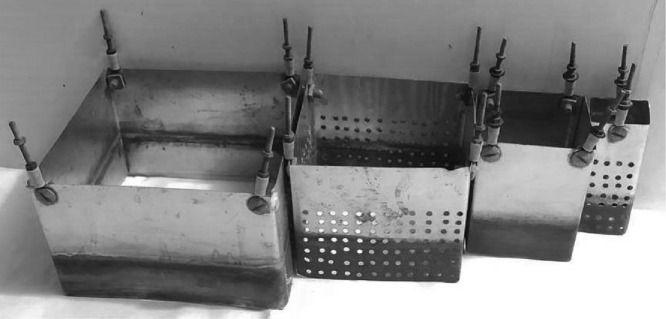

Specifications table

**Table:** 

Subject Area:	Water and wastewater treatment
More specific subject area:	Toxic heavy metals
Method name:	Electrocoagulation removal of Pb, Cd, and Cu ions from wastewater using a new configuration of electrodes
Name and reference of original method:	Vik, E. A., Carlson, D. A., Eikun, A. S. and Gjessing, E. T. 1984. Electrocoagulation of potable water. Water Res., 18: 1355–1360.
Resource availability:	https://doi.org/10.1016/0043–1354(84)90003–4

## Method details

### Overview

In recent decades, rapid industrialization and modern agricultural practices, as well as unplanned urbanization, have affected the environment with numerous contaminants that threaten humanity [1], [2], [3], [4]. Water contamination by heavy metals produced from several activities, such as metal plating, ore mining, fertilizer, batteries, paper, paints, pesticides, etc. (Table 1), has widely received the attention of many scientists and researchers. A batch EC containing bipolar aluminum electrodes was used by Assadi, et al. (2015) to remove lead from wastewater under the effect of the electrolysis time (5–30 min) and current density (11, 22, 33 A/m2), lead concentrations (5–15) ppm, and pH (5–9). The highest removal of lead attained was 94% at pH 7, 33 A\m2 of current density, and 30 min of the reaction time [5]. Abdul Rehman, et al. (2015) conducted a continuous EC reactor containing bipolar aluminum and iron plane electrodes to eliminate 105 ppm of Cu, 110 ppm of Ni, and 63 ppm of Pb from wastewater under the impact of current density (0.0070–0.040) A/cm2, retention time (20–120 s), pH (3–9) and (4–24 mm) of the distance between electrodes. This work attained 95% of metals removal efficiency, 0.026 A/cm2 of the current density, and a solution pH of 6.32 [6]. Al-Nuaimi and Pak (2016) attained 91.72% of chromium removal efficiency at 14 mA/cm2 of current density, pH =6.7, 1 cm of the distance between electrodes 15 g/l of KCl, and 90 min of reaction time [7]. Abdul Majeed, et al. (2018) employed a batch EC reactor containing two aluminum electrodes as the anode and copper-cathode to eliminate nickel from wastewater under the influences of voltages and the reaction time. They achieved the highest removal of Ni at 5 Vs within 76 min [8]. While Patel and Parikh (2020) removed chromium (VI) from wastewater using copper electrodes in an EC reactor. The highest removal was 98.15% at 41.32 A/m2 of current density, pH 7, and 1.4 cm of the distance between the electrodes [9]. It will show later other previous studies whose concerned with removing heavy metals from wastewater in a summarized table.

**Table 1:** The main sources of heavy metal contamination in the environment [13].

Sources	Domestic	Industries	Agriculture
Activities	• Detergents • Organic and inorganic waste • Medical utilities • Batteries discharged	• Mining and metal producing • Combustion of fuels	• Sewage sludge applied in agriculture. • Pesticides and organic fertilizer

Heavy metals are non- degradable and not converted to more simple forms, like organic pollutants. They are also toxic due to their effects on living organisms, easy bioaccumulation in the food chain, and comprehensive sources [1,^2^,10]. The wastewater is classified as water-toxic metal pollution when it is polluted by high-density elements with (63.5 to 200.6) atomic weight or an atomic number greater than 20 and a weight density greater than 5 g/cm^3^ [1], [2], [3],11]. Heavy metals such as, lead (Pb), cadmium (Cd), zinc (Zn), chromium (Cr), copper (Cu), mercury (Hg), nickel (Ni), manganese (Mn), silver (Ag), platinum (Pt), and arsenic (As) are released daily from different industrial and domestic activities, causing a serious hazard to the human health, natural plant, and fauna [2,[12], [13], [14]. Table 2 shows the major sources, health effects of some toxic metals that have been treated in the present work, and their permission limits on drinking water as documented by the World Health Organization (WHO) [1,^13^,15].

**Table 2:** The main effects, sources, and permission limits of some toxic metals [1,[13], [14], [15].

Heavy metals	Sources	Toxicity influences on health (mg per kg of body weight)	Natural water (µg/L)	Fresh water (µg/L)	Agricultural water (µg/L)	Drinking water WHO (µg/L)
Pb	-Purifying of metal -Emissions from different vehicles -Agriculture fertilizers and pesticides. - Digital products and batteries. -Combustion of leaded fuels such as gasoline.	- Kidney failure. - Gastric and lung cancer - Brain tumors. - Deposition in the bones. (**Toxicity: 0.025 weekly**)	0.007–308	0.34	5000	10
Cd	– Electroplating and Metallurgical industries – Some petroleum products – Some types of insecticides	- Kidney failure. - Liver damage. - Anemia. - Bone degeneration. - Carcinogenesis. (**Toxicity: 0.025 monthly**)	6 × 10^−4^–0.61	0.08–0.25	10	3
Cu	– Waste discharges from industries. – Metal alloys pigments – Electroplating and mining –Combustion of coal	- Anemia. - Pain - Inflammation of skin. (**Toxicity: 1 daily**)	0.23–2.59	8.2	200	2000

As revealed in Table 2, toxic metals must be eliminated from water using effective treatment methods before being discharged into the environment because they are non-biodegradable compounds.

### Treatment methods for heavy metal removal

Different technologies have been conducted individually and/or in combination systems to eliminate toxic metals from wastewater [16], such as nanofiltration [17], adsorption [18], bioremediation [19], flocculation [20], chemical precipitation [21], ion exchange [22], nanomaterials [23], and microbial electrolysis cells [24]. Moreover, electrochemical technologies have been performed to treat metal wastewater, such as electrooxidation [25], Electro-peroxone [26], electroflotation [27], electrodeposition [28], electroflocculation and electroreduction [29], electrocoagulation and adsorption [16], and Peroxi-coagulation [30]. Electrochemical technologies overcome many drawbacks observed when conventional treatment methods are employed to remove heavy metals from different sources of wastewaters [31].

### Electrocoagulation

**Fig. 1:**
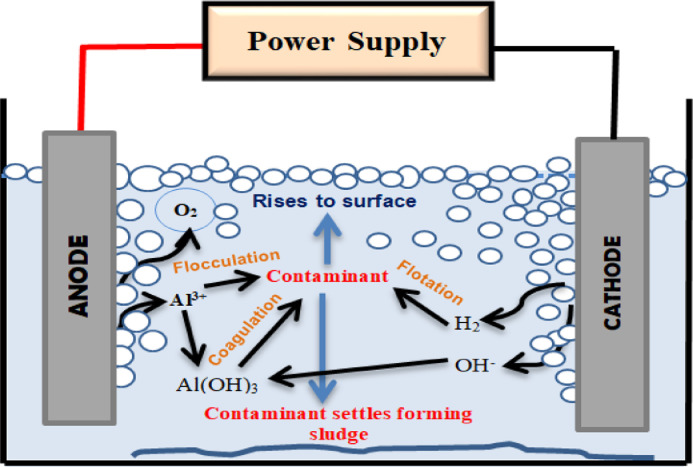
General schematic of electrocoagulation technique.

Electrocoagulation (EC) is a type of electrochemical technique including essential advantages, such as versatility, cost-effectiveness, selectivity, safety, low value of sludge production, considerable removal efficiency, and energy efficiency [32], [33], [34]. The mechanism of EC depends on the electrochemical production of metallic ions from electrodes (such as aluminum that is used in this investigation) to form coagulants (Aluminum hydroxide Al(OH)3) which are required to remove pollutants from samples Fig. 1). Eqs. (1)–(3) show the chemical reactions occur in the EC reactor at the anode and cathode [32,^35^,36]:

(1) At the anode: Al_(S)_ ⇒ Al^3+^_(aq)_ + 3e^−^

(2) At the cathode: 2H_2_O + 2e^−^ ⇒ H_2(g)_ + 2OH^−^_(aq)_

(3) Formation of coagulants: Al^3+^ +3OH^−^⇔ Al(OH)_3_

Removal efficiencies (Y_i_) of heavy metals, i.e. (Y_Pb_%), (Y_Cd_%), and (Y_Cu_%), were estimated using Eq. (4) as follows [37]:

(4) $$Y_{i}\%=\;\frac{C_{0}-C_{t}}{C_{0}}\;\times \;100$$

where *C_0_* and *C_t_* are the concentrations of metal at time (0) and (t), respectively.

In addition, the consumption of energy (kWh/m^3^) and the theoretical consumption of electrodes were measured by using the Eqs. (5) and (6) [31,^32^,36]:

(5) $$ENC=\frac{VIt}{V_{R}}$$

where *V* is the cell voltage in volt, *I* will be the current intensity in ampere, *t* is the reaction time in hour, and *V_R_* is the volume of wastewater sample in cubic meter.

(6) $$TC=\frac{ItM}{ZF}$$

where *M* is the M.wt of aluminum electrodes, *Z* equals 3 (for aluminum), and *F* is Faraday's constant, which equals 96,485.34 Columb/mol.

The actual consumption of aluminum electrodes is determined depending on the weight difference of aluminum electrodes before and after each run.

Hence, the work aims to assess the multi-heavy metal removal efficiency and determine the consumption of energy and electrodes under the impact of specific operating conditions by employing a new configuration of multi-concentric-cubic aluminum electrodes. The reactor shape and the configuration of the electrodes are the keys to estimating the performance of each electrocoagulation reactor [31]. The response surface methodology type Box-Behnken design (RSM-BBD) and Minitab soft program were used for experimental design and analysis.

## Experimental work

### Apparatus

**Fig. 2:**
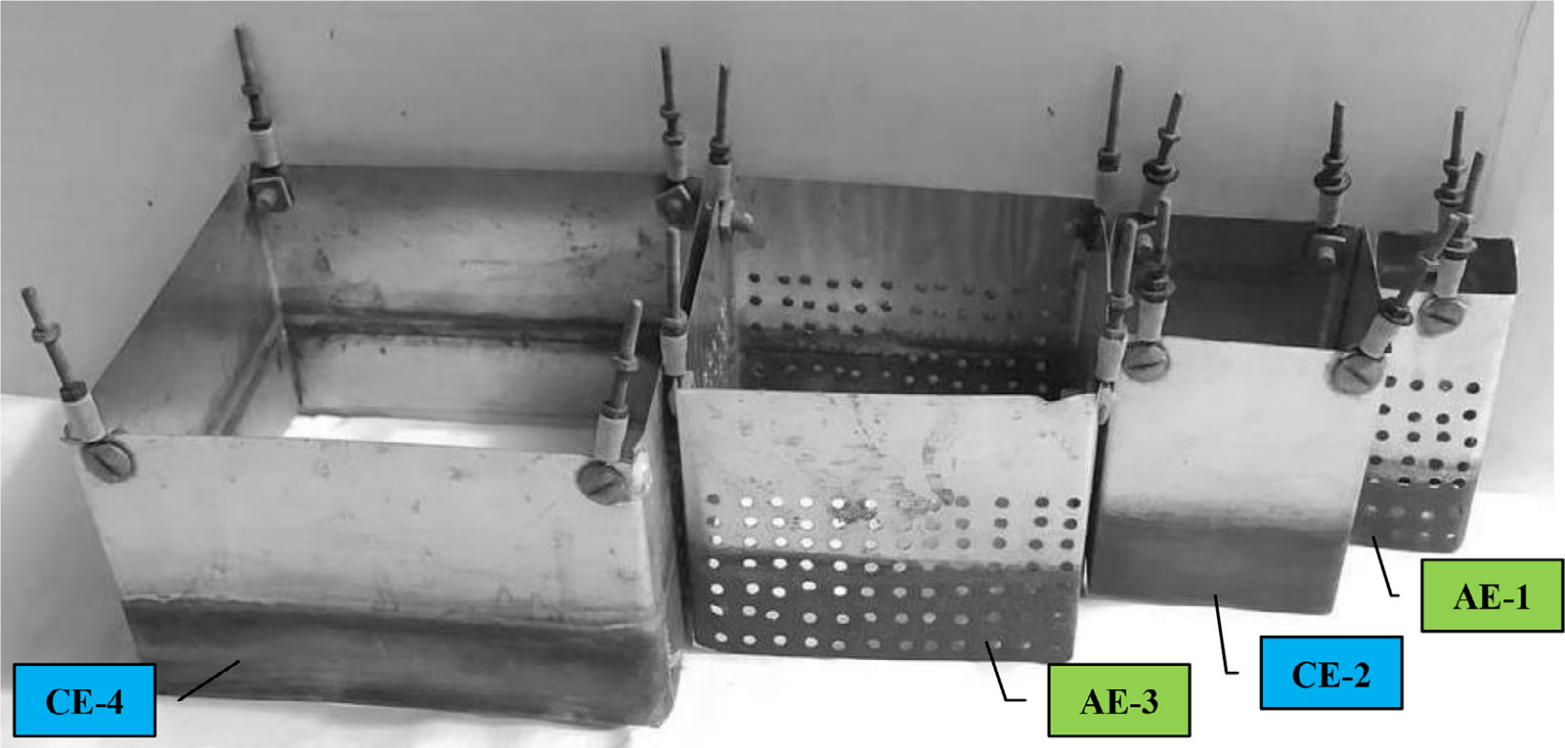
The configuration of the cubic electrodes.

Four concentric aluminum cubic electrodes with different dimensions (Fig. 2 and Table 3) are employed in a batch EC reactor. The anode electrodes (AE-1 and AE-3) have been perforated with a total active area of 360 cm^2^, while the cathode electrodes (CE-2 and CE-4) were non-perforated with a total active area of 512 cm^2^.

**Table 3:** The dimensions of the cubic aluminum electrodes.

Electrode No.	Anode/ Cathode	Plane/ Perforated	Dimensions (cm)	Electrode thickness (cm)	Distance of electrodes (cm)	Wet height (cm)	Active area (cm^2^)
AE-1	Anode	Perforated	4 × 4 × 10	0.2	2	4	50
CE-2	Cathode	Non-Perforated	8 × 8 × 10	0.2	2	4	256
AE-3	Anode	Perforated	12 × 12 × 10	0.2	2	4	310
CE-4	Cathode	Non-Perforated	16 × 16 × 10	0.2	2	4	256

However, the XRD-test result of aluminum electrodes is shown in Table 4 as follows:

**Table 4:** XRD- test analysis of the present electrodes.

Metals	Al	Fe	Mg	C	Si
% Weight	86.91	0.08	0.77	11.55	0.69

This study has conducted the EC experiments at room temperature using a glass cell with a total volume of 3000 ml and 2000 ml of the experimental volume. It provided a constant stirring speed of 300 rpm for the EC reactor containing solution using a magnetic stirrer (Model: ALFA company: HS-860; 0–3000 rpm). The electrodes were connected to a DC-power supply (Model: DC power supply type SYADGONG, China) and arranged in a bipolar connection mode. Solution pH was adjusted to the designed value using 0.1 N HCl and 0.1 N NaOH. Hence, the value of pH was measured before and after each experiment using an electronic pH meter (Model: ATC company, China). The electrolyte used for support was an NaCl solution of 2 g/L. The synthetic wastewater was prepared by dissolving the design amounts of [Pb(NO_3_)_2_: 95% purity], (Cd(NO_3_)_2__·_4H_2_O: 95% purity), and [Cu(NO_3_)_2_·3H_2_O: 95% purity] salts in the supporting electrolyte.

After each run, these electrodes were polished with soft sandpaper and washed with dilute HCl and distillate water to remove the oxide layer, and then dried to be weighed after electrocoagulation. Final concentrations of toxic metals were determined using atomic absorption spectroscopy (type-AA-7000F, Shimadzu, Japan) and the removal efficiency of these metals was calculated using Eq. (4).

### Experimental design

To approach a few experimental processes involving the interaction of the studied variables and modeling of parameters of the studied responses, response surface methodology (RSM) type Box–Behnken Design (BBD) and Minitab program were used to design the EC experiments and analyze the results.

In this investigation, RSM-BBD optimized operating variables of Pb initial concentration (X1: 0–200 ppm), Cd initial concentration (X2: 0–200 ppm), Cu initial concentration (X3: 0–200 ppm), solution pH (X4: 4–10), applied current (X5: 0.2–2.6 A) and reaction time (X6: 4–60 min) to maximize the removal efficiencies of metals and minimize consumption of energy and electrodes. The real and coded values of the operating variables are listed in Table 5 and the experimental design matrix from RSM-BBD is explained in Table 6, which comprises 52 actual conditions and the core results of the studied responses.

**Table 5:** Real and Coded variables of the operating variables.

Variables	Units	Levels		
		−1	0	+1
X_1_: Pb concentration	ppm	0	100	200
X_2_: Cd concentration	ppm	0	100	200
X_3_: Cu concentration	ppm	0	100	200
X_4_: pH	–	4	7	10
X_5_: Applied Current	A	0.2	1.4	2.6
X_6_: Reaction time	min	4	32	60

**Table 6:** Removal efficiencies and the consumption of energy and electrodes.

Run	X_1_	X_2_	X_3_	X_4_	X_5_	X_6_	Y_Pb_%	Y_Cd_%	Y_Zn_%	Real Cons. of AE-1 (g)	Real Cons. of AE-3 (g)	Real Cons. of Anodes (g)	Theoretical Cons. of Anodes (g)	ENC (kWh/m^3^)	Final
	(ppm)	(ppm)	(ppm)	–	(A)	(min)									pH
1	0	0	100	4	1.4	32	0.00	0.00	98.54	0.07	0.78	0.85	0.251	5.35	6.7
2	200	0	100	4	1.4	32	99.70	0.00	99.57	0.49	0.44	0.93	0.251	8.33	5.6
3	0	200	100	4	1.4	32	0.00	80.21	78.96	0.90	0.17	1.07	0.251	7.68	5.9
4	200	200	100	4	1.4	32	93.14	60.08	94.92	0.12	0.15	0.27	0.251	7.35	7.5
5	0	0	100	10	1.4	32	0.00	0.00	94.72	0.41	0.04	0.45	0.251	7.23	8.6
6	200	0	100	10	1.4	32	95.38	0.00	94.79	0.39	0.20	0.59	0.251	8.21	8.7
7	0	200	100	10	1.4	32	0.00	97.00	88.57	0.25	0.75	1.00	0.251	4.33	8.5
8	200	200	100	10	1.4	32	99.33	99.86	99.23	0.09	0.18	0.27	0.251	5.70	9.6
9	100	0	0	7	0.2	32	93.28	0.00	0.00	0.04	0.09	0.13	0.036	0.30	7.5
10	100	200	0	7	0.2	32	91.88	59.11	0.00	0.06	0.06	0.12	0.036	0.29	6.5
11	100	0	200	7	0.2	32	98.90	0.00	99.80	0.92	0.41	1.33	0.036	0.20	9.2
12	100	200	200	7	0.2	32	80.12	83.07	97.58	0.17	0.01	0.18	0.036	0.26	6.4
13	100	0	0	7	2.6	32	97.50	0.00	0.00	0.21	0.26	0.47	0.465	16.09	7.7
14	100	200	0	7	2.6	32	98.12	77.91	0.00	0.49	0.42	0.91	0.465	16.53	8.7
15	100	0	200	7	2.6	32	95.68	0.00	98.56	0.21	0.28	0.49	0.465	16.03	9.1
16	100	200	200	7	2.6	32	94.57	86.53	97.62	0.29	0.18	0.47	0.465	16.31	8.7
17	100	100	0	4	1.4	4	26.04	17.25	0.00	0.18	0.02	0.20	0.031	1.08	4.3
18	100	100	200	4	1.4	4	33.83	5.83	98.46	0.05	0.05	0.10	0.031	1.10	5.0
19	100	100	0	10	1.4	4	87.55	85.58	0.00	0.07	0.06	0.13	0.031	1.05	9.5
20	100	100	200	10	1.4	4	80.57	71.52	96.56	0.07	0.06	0.13	0.031	1.09	9.8
21	100	100	0	4	1.4	60	96.92	91.77	0.00	0.27	0.29	0.56	0.470	12.82	6.1
22	100	100	200	4	1.4	60	99.33	98.69	99.93	0.67	0.22	0.89	0.470	12.49	8.8
23	100	100	0	10	1.4	60	98.82	97.65	0.00	0.30	0.19	0.49	0.470	12.15	8.4
24	100	100	200	10	1.4	60	99.73	98.54	98.93	0.24	0.31	0.55	0.470	12.71	8.3
25	0	100	100	4	0.2	32	0.00	28.42	35.52	0.04	0.03	0.07	0.036	0.20	5.3
26	200	100	100	4	0.2	32	38.19	31.57	36.95	0.02	0.03	0.05	0.036	0.21	5.5
27	0	100	100	10	0.2	32	0.00	98.45	99.41	0.02	0.07	0.09	0.036	0.22	9.5
28	200	100	100	10	0.2	32	99.53	98.05	99.66	0.06	0.06	0.12	0.036	0.23	9.6
29	0	100	100	4	2.6	32	0.00	81.48	92.77	0.34	0.29	0.63	0.465	16.03	6.9
30	200	100	100	4	2.6	32	95.33	86.02	95.21	0.25	0.29	0.54	0.465	15.48	7.7
31	0	100	100	10	2.6	32	0.00	98.78	99.68	0.45	0.30	0.75	0.465	14.75	8.2
32	200	100	100	10	2.6	32	98.24	99.25	99.84	0.34	0.21	0.55	0.465	15.14	8.1
33	100	0	100	7	0.2	4	25.18	0.00	43.26	0.02	0.03	0.05	0.004	0.02	7.2
34	100	200	100	7	0.2	4	10.43	32.51	41.56	0.03	0.04	0.07	0.004	0.03	7.7
35	100	0	100	7	2.6	4	61.72	0.00	70.65	0.09	0.04	0.13	0.058	1.92	8.0
36	100	200	100	7	2.6	4	59.90	50.76	63.02	0.70	0.06	0.76	0.058	1.95	7.4
37	100	0	100	7	0.2	60	72.35	0.00	89.25	0.05	0.04	0.09	0.067	0.54	8.2
38	100	200	100	7	0.2	60	77.01	78.44	86.45	0.02	0.06	0.08	0.067	0.74	8.8
39	100	0	100	7	2.6	60	97.07	0.00	95.49	0.43	0.48	0.91	0.873	24.75	9.1
40	100	200	100	7	2.6	60	84.00	89.33	94.56	0.40	0.51	0.91	0.873	27.77	8.2
41	0	100	0	7	1.4	4	0.00	74.95	0.00	0.03	0.07	0.10	0.031	1.06	7.8
42	200	100	0	7	1.4	4	61.55	11.44	0.00	0.09	0.06	0.15	0.031	0.91	7.4
43	0	100	200	7	1.4	4	0.00	77.41	88.86	0.05	0.21	0.26	0.031	0.84	8.1
44	200	100	200	7	1.4	4	27.55	10.55	50.78	0.02	0.03	0.05	0.031	0.93	8.7
45	0	100	0	7	1.4	60	0.00	94.70	0.00	0.35	0.24	0.59	0.470	15.29	9.5
46	200	100	0	7	1.4	60	96.67	91.87	0.00	0.30	0.42	0.72	0.470	13.33	8.3
47	0	100	200	7	1.4	60	0.00	96.53	98.84	0.31	0.22	0.53	0.470	12.94	8.9
48	200	100	200	7	1.4	60	97.72	90.20	97.52	0.49	0.40	0.89	0.470	12.26	8.4
49	100	100	100	7	1.4	32	93.00	97.65	97.77	0.28	0.18	0.46	0.251	7.08	8.4
50	100	100	100	7	1.4	32	92.75	97.23	97.04	0.26	0.22	0.48	0.251	6.84	7.8
51	100	100	100	7	1.4	32	95.10	98.40	98.01	0.21	0.19	0.40	0.251	6.78	7.7
52	100	100	100	7	1.4	32	95.67	98.47	97.54	0.18	0.23	0.41	0.251	7.23	8.2

## Results and discussions

### Removal efficiency of Pb, Cd, and Cu metals

Table 6 listed the removal efficiencies of toxic metals based on the aluminum-concentric cubic electrodes and considered all factors. In Table 6, columns 2, 3, 4, 5, 6, and 7 indicate the actual values of the operating variables; initial concentrations of Pb, Cd, and Cu metals (ppm), solution pH, electric current (A), and the reaction time (min). The NaCl electrolyte was used to assist in the removal of toxic metals from synthetic wastewater. The EC technology is dependent on the solution pH of the wastewater during the periods of the experiments. Solution pH influences the formation of metallic electro-coagulants, and the initial solution pH influences the EC performance [32,[38], [39], [40]. The type of electrode material, especially the anode, affects the performance of the electrocoagulation reactor because it determines the kind of cations that are released into the solution that plays a significant role in the formation of flocs [32].

**Fig. 3:**
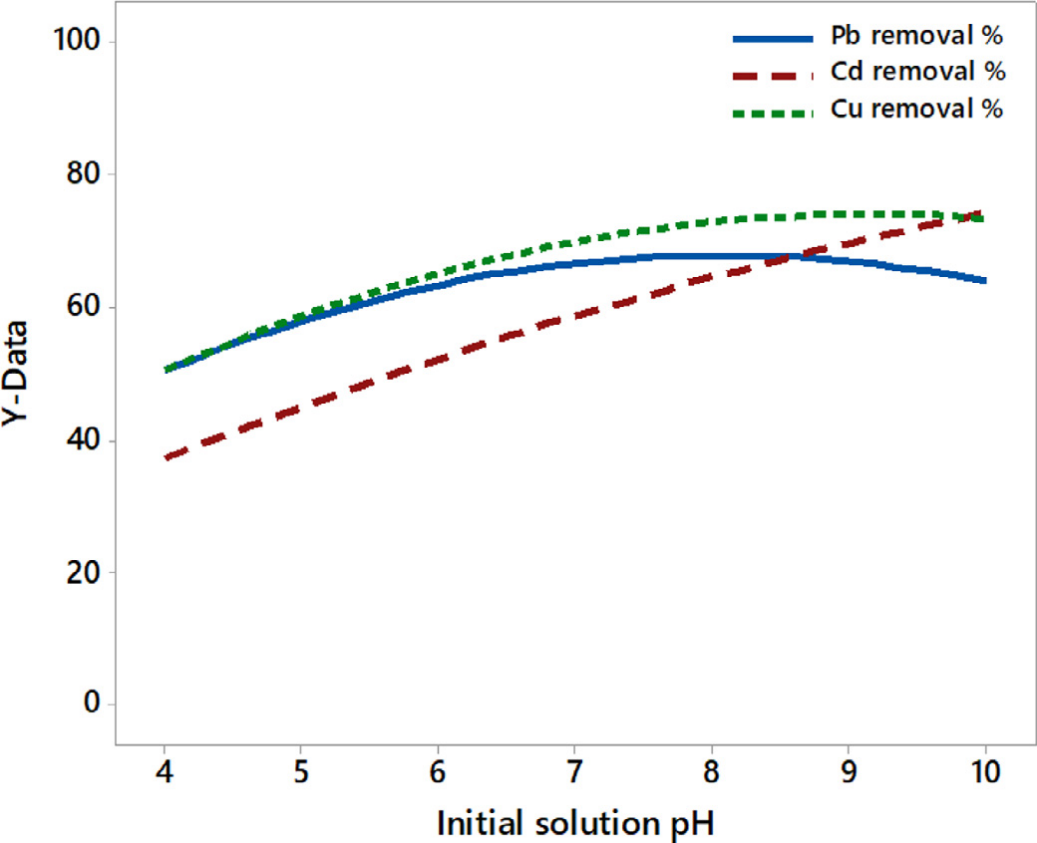
The effect of solution pH on the removal efficiency of toxic metals.

As shown in Fig. 3, the removal efficiencies are increased when the initial pH of wastewater is increased until the higher value of pH because of the formation of undesired hydroxo-complexes such as [Al(OH)_4_^−^] and [Al(OH)_5_^2−^] which are not useful to form electro-coagulants and then affect the treatment process [41]. However, the Cd removal efficiency kept increasing even in the higher basic solution. These results agreed with [15,^32^,42], which indicated the significant effect of solution pH value on the removal efficiency of heavy metals.

It is evident that the removal efficiency of each metal differs in its behavior based on solution pH, which is associated with the formation of hydroxyl ions at the cathode and Al ions from the anode as a natural result of the continuous passing of electricity through the aluminum electrodes. The formation of electro-coagulants on one side and the variation in the pH value on the other will influence the metal removal efficiency value. The difference in the current value supplied to the EC cell during the electrocoagulation process of each experiment clearly established this.

The mathematical equations of curves presented in Fig. 3 that relate removal efficiencies to the initial value of solution pH (X_4_) are as follows Eqs. (7)–(9):

(7) $$Y_{\text{Pb}\%}=16.83\;X_{4}-1.042\;X_{4}^{2}\left(100\;\text{ppm}\;\text{Pb};100\;\text{ppm}\;\text{Cd},\;100\;\text{ppm}\;\text{Cu};\;1.4\;A;\;32\;\min\right)$$

(8) $$Y_{\text{Cd}\%}=10.67\;X_{4}-0.323\;X_{4}^{2}\left(100\;\text{ppm}\;\text{Pb};100\;\text{ppm}\;\text{Cd},\;100\;\text{ppm}\;\text{Cu};\;1.4\;A;\;32\;\min\right)$$

(9) $$Y_{\text{Cu}\%}=16.18\;X_{4}-0.883\;X_{4}^{2}\;\left(100\;\text{ppm}\;\text{Pb};100\;\text{ppm}\;\text{Cd},\;100\;\text{ppm}\;\text{Cu};\;1.4\;A;\;32\;\min\right)$$

**Fig. 4:**
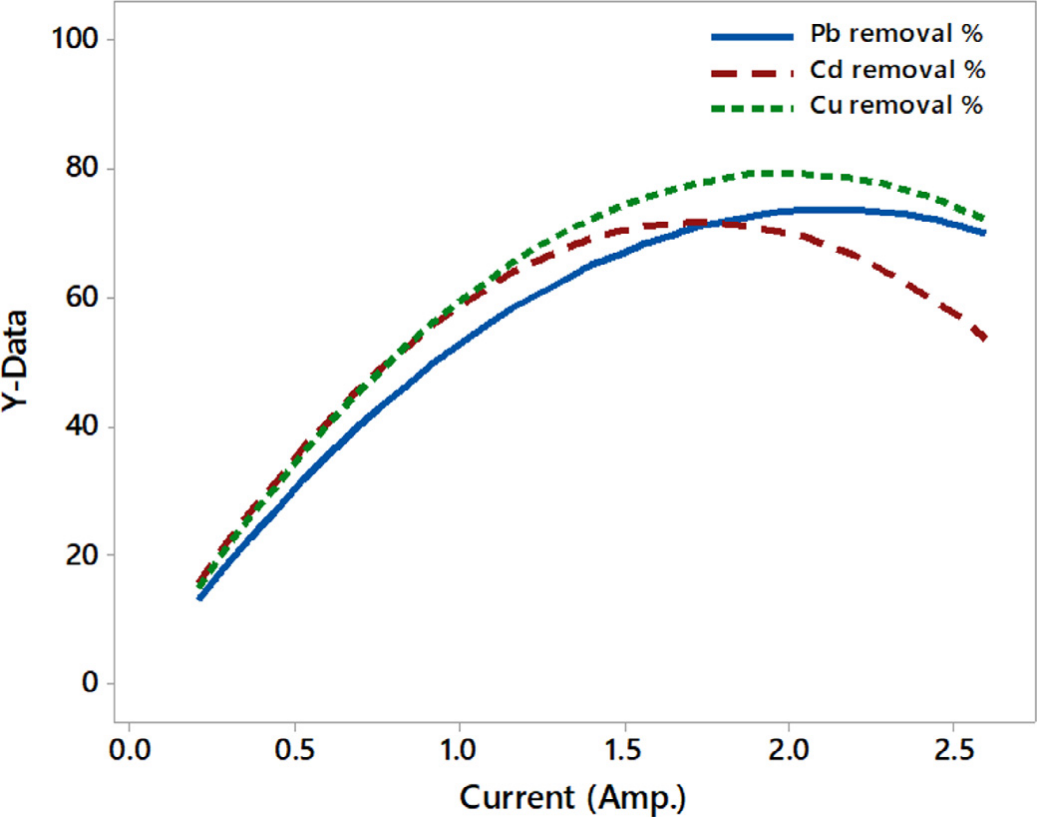
The effect of applied current on the removal efficiency of toxic metals.

The current intensity has a significant impact on the EC process because it regulates the formation of electro-coagulants depending on the anodic dissolution based on Faraday's law. It is the main parameter of the performance of all electrochemical methods, which is the more effective parameter to control the rate of reaction in the electrochemical cell. Therefore, the removal efficiencies of toxic metals are increased when the current intensity is raised [43], [44], [45]. The influence of applied electric current on the EC cell is especially essential since the release of hydrogen and oxygen rate influences the mechanism of the EC process [46], [47], [48]. As observed, when the applied current raised from 0.2 to 2.6 A, toxic metals elimination increased, as shown in Fig. 4 and their mathematical relations Eqs. (10)–(12). The excessive increase of current will increase the ohmic drop and subsequently affect the EC performance. As seen in Fig. 4, the Cd metal was more sensitive to the continuous increase of applied current compared to the other two metals that kept eliminated until a higher value of current. These findings are the same as [42,^49^,50]. It is obvious that the continuous supplying of electric current through electrodes has to control the amount of metals ions released from them and, consequently, enhancing the removal efficiencies of metals until the optimal conditions.

(10) $$Y_{\text{Pb}\%}=69.21X_{5}-16.24\;X_{5}^{2}\;\left(100\;\text{ppm}\;\;\text{Pb};100\;\text{ppm}\;\text{Cd},\;100\;\text{ppm}\;\text{Cu};\;\text{pH}\;7;\;32\;\min\right)$$

(11) $$Y_{\text{Cd}\%}=82.85\;X_{5}-23.91\;X_{5}^{2}\;\left(100\;\text{ppm}\;\text{Pb};100\;\text{ppm}\;\text{Cd},\;100\;\text{ppm}\;\text{Cu};\;\text{pH}\;7;\;32\;\min\right)$$

(12) $$Y_{\text{Cu}\%}=79.63\;X_{5}-19.96\;X_{5}^{2}\;\left(100\;\text{ppm}\;\text{Pb};100\;\text{ppm}\text{Cd},\;100\;\text{ppm}\;\text{Cu};\;\text{pH}\;7;\;32\;\min\right)$$

**Fig. 5:**
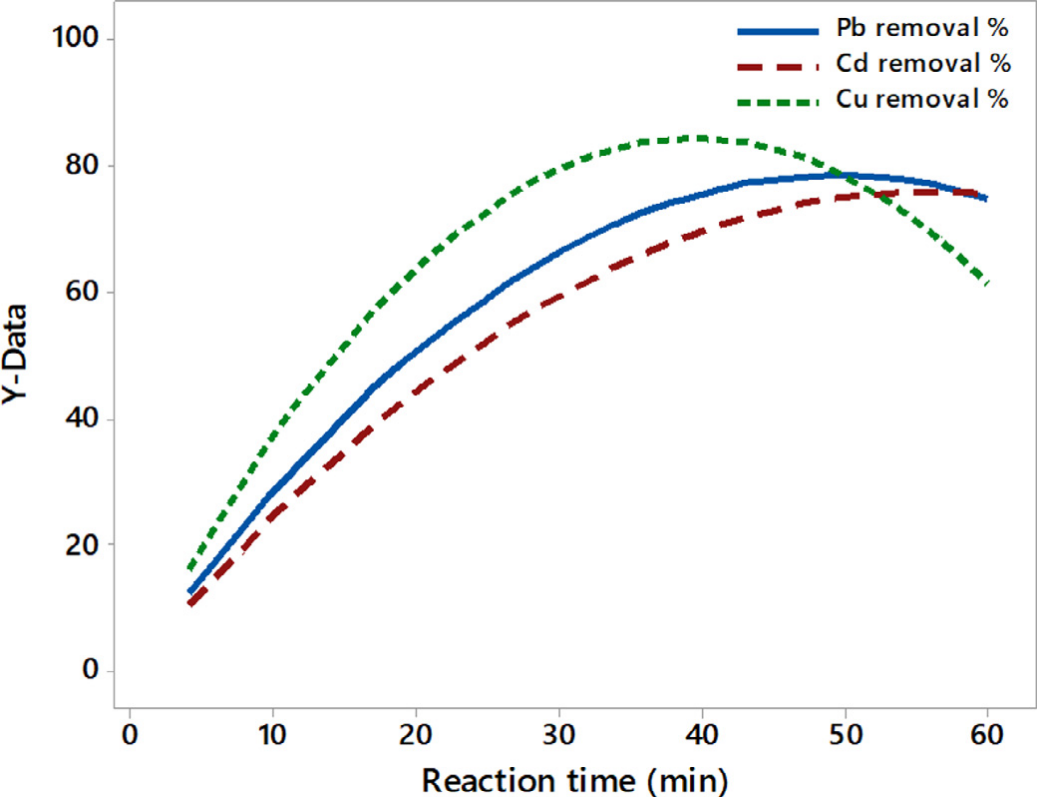
The effect of the reaction time on the removal efficiency of toxic metals.

The operating variable of the reaction time is extremely affecting the quantity of Al released from the concentric electrodes, which react with OH ions to form the electro-coagulants that determine the toxic metals’ removal efficiencies [51], [52], [53]. Fig. 5 reveals the effect of these variables on the removal efficiencies where all metals have removed when the reaction time increased, but Cu removal was rapidly increased and then early decreased compared to other metals that have minimized after a while. This behavior depends on the ability of the attraction force between each metal and electro-coagulants formed throughout the reactor. These results are like those of [4,^32^,35]. The mathematical correlations between the removal efficiencies and the reaction time (X_6_) are as follows Eqs. (13)–((15)):

(13) $$Y_{\text{Pb}\%}=3.18\;X_{6}-0.03\;X_{6}^{2}\;\left(100\;\text{ppm}\;\text{Pb};100\;\text{ppm}\;\text{Cd},\;100\;\text{ppm}\;\text{Cu};\;\text{pH}\;7;\;1.4\;A\right)$$

(14) $$Y_{\text{Cd}\%}=2.71\;X_{6}-0.02\;X_{6}^{2}\;\left(100\;\text{ppm}\;\text{Pb};100\;\text{ppm}\;\text{Cd},\;100\;\text{ppm}\;\text{Cu};\;\text{pH}\;7;\;1.4\;A\right)$$

(15) $$Y_{\text{Cu}\%}=4.29\;X_{6}-0.05\;X_{6}^{2}\;\left(100\;\text{ppm}\;\text{Pb};100\;\text{ppm}\;\text{Cd},\;100\;\text{ppm}\;\text{Cu};\;\text{pH}\;7;\;1.4\;A\right)$$

The highest removal efficiencies of toxic metals were Pb-99.73%, Cd-98.54%, and Cu-98.92% at pH 10, applied current of 1.4 A, and reaction time of 60 min. The total actual consumption of electrodes under these conditions was 0.55 g and the energy consumption was 12.71 kWh/m^3^. These results show that the EC has attained the maximum removal efficiencies of toxic metals with low consumption of electrodes and electrical energy.

### Analysis with RSM-BBD

The RSM is a statistical method that is useful for designing experiments, analyzing, and optimizing the studied variables and responses [32,^54^,55]. The validation of the adequacy of the mathematical models estimated is achieved by using the analysis of variance (ANOVA). This test is used to analyze the regression models and fit them to the data in order to estimate the misleading findings that may influence the accuracy of the developed regression models [56,57].

Table 7, Table 8, Table 9, Table 10 show the ANOVA test results for Pb removal%, Cd removal%, Cu removal%, and energy consumption. These results show that the studied responses have a significant impact (*p* < 0.05) on the removal of heavy metals, which means that the estimated model is significant at 95% of the probability level. However, Table 11 lists the mathematical models of the studied responses and their regression coefficients, where the highest values of these coefficients mean that the quadratic models are significant [56].

**Table 7:** ANOVA-test results for Pb removal efficiency.

Source	Sum of Squares	DF	Mean Square	F-value	P-value	
Model	82,481.7	27	3054.9	20.34	<0.0001	Highly Significant
X_1_: initial Pb	41,861.2	1	41,861.2	278.66	<0.0001	Highly Significant
X_2_: initial Cd	97.0	1	97.0	0.65	0.429	
X_3_: initial Cu	67.8	1	67.8	0.45	0.508	
X_4_: pH	1300.5	1	1300.5	8.66	0.007	Significant
X_5_: Current	1588.6	1	1588.6	10.58	0.003	Significant
X_6_: Time	8262.3	1	8262.3	55.00	<0.0001	Highly Significant
X_1_^2^	17,780.5	1	17,780.5	118.36	<0.0001	Highly Significant
X_2_^2^	10.9	1	10.9	0.07	0.790	
X_3_^2^	728.5	1	728.5	4.85	0.038	
X_4_^2^	11.4	1	11.4	0.08	0.786	
X_5_^2^	617.4	1	617.4	4.11	0.054	
X_6_^2^	5405.7	1	5405.7	35.98	<0.0001	Highly Significant
X_1_ X_2_	0.9	1	0.9	0.01	0.94	
X_1_ X_3_	135.7	1	135.7	0.90	0.351	
X_1_ X_4_	273.2	1	273.2	1.82	0.19	
X_1_ X_5_	390.0	1	390.0	2.60	0.12	
X_1_ X_6_	1385.7	1	1385.7	9.22	0.006	Significant
X_2_ X_3_	45.7	1	45.7	0.30	0.587	
X_2_ X_4_	13.8	1	13.8	0.09	0.764	
X_2_ X_5_	13.9	1	13.9	0.09	0.764	
X_2_ X_6_	8.30	1	8.30	0.06	0.816	
X_3_ X_4_	33.1	1	33.1	0.22	0.643	
X_3_ X_5_	0.1	1	0.1	0.0	0.983	
X_3_ X_6_	88.2	1	88.2	0.59	0.451	
X_4_ X_5_	426.7	1	426.7	2.84	0.105	
X_4_ X_6_	1403.3	1	1403.3	9.34	0.005	Significant
X_5_ X_6_	368.5	1	368.5	2.45	0.13	
Residual	3605.4	24	150.2			
Lack of Fit	3598.8	21	171.4	78.85	0.002	
Pure Error	6.5	3	2.2			
Total	86,087.0	51

**Table 8:** ANOVA-test results for Cd removal efficiency.

Source	Sum of Squares	DF	Mean Square	F-value	P-value	
Model	80,403.0	27	2977.9	14.27	<0.0001	Highly Significant
X_1_: initial Pb	925.6	1	925.6	4.43	0.046	Significant
X_2_: initial Cd	33,363.5	1	33,363.5	159.85	<0.0001	Highly Significant
X_3_: initial Cu	11.5	1	11.5	0.06	0.816	
X_4_: pH	5501.5	1	5501.5	26.36	<0.0001	Highly Significant
X_5_: Current	1072.6	1	1072.6	5.14	0.033	Significant
X_6_: Time	10,000.5	1	10,000.5	47.91	<0.0001	Highly Significant
X_1_^2^	487.7	1	487.7	2.34	0.139	
X_2_^2^	17,997.7	1	17,997.7	86.23	<0.0001	Highly Significant
X_3_^2^	551.5	1	551.5	2.64	0.117	
X_4_^2^	217.2	1	217.2	1.04	0.318	
X_5_^2^	626.6	1	626.6	3.00	0.096	
X_6_^2^	2004.9	1	2004.9	9.61	0.005	Significant
X_1_ X_2_	37.3	1	37.3	0.18	0.676	
X_1_ X_3_	5.9	1	5.9	0.03	0.868	
X_1_ X_4_	14.8	1	14.8	0.07	0.793	
X_1_ X_5_	0.6	1	0.6	0.00	0.956	
X_1_ X_6_	1836.3	1	1836.3	8.80	0.007	Significant
X_2_ X_3_	132.7	1	132.7	0.64	0.433	
X_2_ X_4_	400.0	1	400.0	1.92	0.179	
X_2_ X_5_	165.2	1	165.2	0.79	0.382	
X_2_ X_6_	892.5	1	892.5	4.28	0.050	
X_3_ X_4_	9.4	1	9.4	0.04	0.834	
X_3_ X_5_	29.4	1	29.4	0.14	0.711	
X_3_ X_6_	63.5	1	63.5	0.30	0.586	
X_4_ X_5_	1404.1	1	1404.1	6.73	0.016	
X_4_ X_6_	2056.9	1	2056.9	9.85	0.004	Significant
X_5_ X_6_	6.8	1	6.8	0.03	0.859	
Residual	5009.3	24	208.7			
Lack of Fit	5008.2	21	238.5	658.43	0.000	
Pure Error	1.1	3	0.4			
Total	85,412.2	51

**Table 9:** ANOVA-test results for Cu removal efficiency.

Source	Sum of Squares	DF	Mean Square	F-value	P-value	
Model	80,825.3	27	2993.5	15.29	<0.0001	Highly Significant
X_1_: initial Pb	2.3	1	2.3	0.01	0.915	
X_2_: initial Cd	74.0	1	74.0	0.38	0.544	
X_3_: initial Cu	52,589.6	1	52,589.6	268.53	<0.0001	Highly Significant
X_4_: pH	823.3	1	823.3	4.20	0.051	
X_5_: Current	1319.4	1	1319.4	6.74	0.016	Significant
X_6_: Time	1799.2	1	1799.2	9.19	0.006	Significant
X_1_^2^	288.1	1	288.1	1.47	0.237	
X_2_^2^	0.1	1	0.1	0.00	0.985	
X_3_^2^	12,817.9	1	12,817.9	65.45	<0.0001	Highly Significant
X_4_^2^	27.0	1	27.0	0.14	0.714	
X_5_^2^	1213.8	1	1213.8	6.20	0.020	Significant
X_6_^2^	1613.4	1	1613.4	8.24	0.008	Significant
X_1_ X_2_	81.4	1	81.4	0.42	0.525	
X_1_ X_3_	194.1	1	194.1	0.99	0.329	
X_1_ X_4_	5.9	1	5.9	0.03	0.863	
X_1_ X_5_	0.1	1	0.1	0.00	0.981	
X_1_ X_6_	168.9	1	168.9	0.86	0.362	
X_2_ X_3_	1.2	1	1.2	0.01	0.937	
X_2_ X_4_	63.5	1	63.5	0.32	0.575	
X_2_ X_5_	0.5	1	0.5	0.00	0.961	
X_2_ X_6_	3.9	1	3.9	0.02	0.889	
X_3_ X_4_	1.1	1	1.1	0.01	0.942	
X_3_ X_5_	0.2	1	0.2	0.00	0.976	
X_3_ X_6_	229.1	1	229.1	1.17	0.290	
X_4_ X_5_	1654.8	1	1654.8	8.45	0.008	Significant
X_4_ X_6_	0.1	1	0.1	0.00	0.982	
X_5_ X_6_	148.7	1	148.7	0.76	0.392	
Residual	4700.2	24	195.8			
Lack of Fit	4699.7	21	223.8	1306.49	0.000	
Pure Error	0.5	3	0.2			
Total	85,525.6	51

**Table 10:** ANOVA-test results for Energy consumption.

Source	Sum of Squares	DF	Mean Square	F-value	P-value	
Model	2536.35	27	93.94	115.24	<0.0001	Highly Significant
X_1_: initial Pb	0.19	1	0.19	0.24	0.630	
X_2_: initial Cd	0.00	1	0.00	0.00	0.995	
X_3_: initial Cu	0.58	1	0.58	0.71	0.406	
X_4_: pH	1.17	1	1.17	1.44	0.242	
X_5_: Current	1342.66	1	1342.66	1647.09	<0.0001	Highly Significant
X_6_: Time	885.86	1	885.86	1086.71	<0.0001	Highly Significant
X_1_^2^	0.24	1	0.24	0.29	0.596	
X_2_^2^	0.19	1	0.19	0.24	0.631	
X_3_^2^	2.79	1	2.79	3.42	0.077	
X_4_^2^	0.48	1	0.48	0.58	0.452	
X_5_^2^	7.06	1	7.06	8.66	0.007	Significant
X_6_^2^	2.27	1	2.27	2.78	0.109	
X_1_ X_2_	1.07	1	1.07	1.31	0.264	
X_1_ X_3_	0.29	1	0.29	0.35	0.557	
X_1_ X_4_	0.03	1	0.03	0.03	0.861	
X_1_ X_5_	0.00	1	0.00	0.00	0.944	
X_1_ X_6_	0.83	1	0.83	1.02	0.322	
X_2_ X_3_	0.00	1	0.00	0.00	0.972	
X_2_ X_4_	5.71	1	5.71	7.01	0.014	Significant
X_2_ X_5_	0.77	1	0.77	0.94	0.341	
X_2_ X_6_	1.26	1	1.26	1.55	0.225	
X_3_ X_4_	0.10	1	0.10	0.13	0.725	
X_3_ X_5_	0.00	1	0.00	0.00	0.954	
X_3_ X_6_	0.58	1	0.58	0.71	0.407	
X_4_ X_5_	0.34	1	0.34	0.42	0.522	
X_4_ X_6_	0.02	1	0.02	0.03	0.874	
X_5_ X_6_	281.08	1	281.08	344.81	<0.0001	Highly Significant
Residual	19.56	24	0.82			
Lack of Fit	19.43	21	0.93	21.02	0.014	
Pure Error	0.13	3	0.04			
Total	2555.91	51

**Table 11:** The quadratic models for the studied responses.

Responses	Models	R^2^	Adjusted R^2^
Pb removal%	Y_Pb Removal__%_ = - 110.8 + 1.004 X_1_ - 0.025 X_2_ - 0.108 X_3_ + 10.91 X_4_+ 36.5 X_5_ + 3.417 X_6_ - 0.004351 X_1_^2^ - 0.000108 X_2_^2^ + 0.000881 X_3_^2^ - 0.122 X_4_^2^ - 5.63 X_5_^2^ - 0.03060 X_6_^2^ - 0.000033 X_1_ X_2_ - 0.000412 X_1_ X_3_ + 0.0138 X_1_×_4_ + 0.0582 X_1_ X_5_ + 0.00470 X_1_×_6_ −0.000239 X_2_ X_3_ + 0.0044 X_2_×_4_ + 0.0078 X_2_×_5_ + 0.00036 X_2_×_6_ - 0.0068 X_3_ X_4_ + 0.0008 X_3_ X_5_ +0.00084 X_3_ X_6_ - 2.03 X_4_ X_5_ - 0.1577 X_4_ X_6_ - 0.202 X_5_ X_6_ (16)	95.81	91.10
Cd removal%	Y_Cd Removal__%_ = - 144.6 - 0.087 X_1_+ 0.906 X_2_ + 0.153 X_3_ + 21.47 X_4_ + 46.8 X_5_ + 2.307 X_6_- 0.000721 X_1_^2^ - 0.004378 X_2_^2^ - 0.000766 X_3_^2^ - 0.534 X_4_^2^ - 5.67 X_5_^2^- 0.01864 X_6_^2^ - 0.000216 X_1_×_2_ - 0.000086 X_1_×_3_+ 0.0032 X_1_×_4_ + 0.0023 X_1_×_5_ + 0.00541 X_1_×_6_+ 0.000407 X_2_×_3_ + 0.0236 X_2_×_4_+ 0.0268 X_2_×_5_+ 0.00377 X_2_×_6_ - 0.0036 X_3_×_4_- 0.0160 X_3_×_5_+ 0.00071 X_3_×_6_ - 3.68 X_4_×_5_ - 0.1909 X_4_ X_6_- 0.027 X_5_ X_6_ (17)	94.14	87.54
Cu removal%	Y_Cu Removal__%_ = - 77.6 + 0.085 X_1_ - 0.116 X_2_ + 1.227 X_3_ + 4.25 X_4_ + 60.5 X_5_+ 1.225 X_6_- 0.000554 X_1_^2^ - 0.000009 X_2_^2^- 0.003694 X_3_^2^+ 0.188 X_4_^2^ - 7.89 X_5_^2^- 0.01672 X_6_^2^+ 0.000319 X_1_×_2_ - 0.000493 X_1_×_3_- 0.0020 X_1_×_4_ + 0.0010 X_1_×_5_ + 0.00164 X_1_×_6_ - 0.000039 X_2_×_3_ + 0.0094 X_2_×_4_ - 0.0014 X_2_×_5_+ 0.00025 X_2_×_6_ - 0.0012 X_3_×_4_ - 0.0013 X_3_×_5_+ 0.00135 X_3_×_6_ - 4.00 X_4_×_5_ + 0.0013 X_4_ X_6_- 0.128 X_5_ X_6_ (18)	94.50	88.32
Energy Consumption	ENC= - 2.75 + 0.0025 X_1_ + 0.0192 X_2_- 0.0145 X_3_+ 0.607 X_4_ - 0.84 X_5_+ 0.0185 X_6_+ 0.000016 X_1_^2^- 0.000014 X_2_^2^ + 0.000055 X_3_^2^- 0.0250 X_4_^2^+ 0.602 X_5_^2^- 0.000626 X_6_^2^ - 0.000037 X_1_×_2_+ 0.000019 X_1_×_3_+ 0.000133 X_1_×_4_ - 0.00019 X_1_×_5_- 0.000115 X_1_×_6_- 0.000001 X_2_×_3_ - 0.00282 X_2_×_4_+ 0.00183 X_2_×_5_+ 0.000142 X_2_×_6_ + 0.00038 X_3_×_4_- 0.00016 X_3_×_5_- 0.000068 X_3_×_6_ - 0.0576 X_4_×_5_- 0.00061 X_4_ X_6_+ 0.17641 X_5_ X_6_ (19)	99.23	98.37

As shown in Tables 7– 9 and based on F-values, the initial concentrations of Pb, Cd, and Cu were the most important variables in Pb, Cd, and Cu removal efficiencies, respectively. While the current applied was the most essential variable in the energy consumption response, as shown in Table 10. The large value F-indicator means that the mean square contributed by the regression model is much higher than the mean square error [57].

**Fig. 6:**
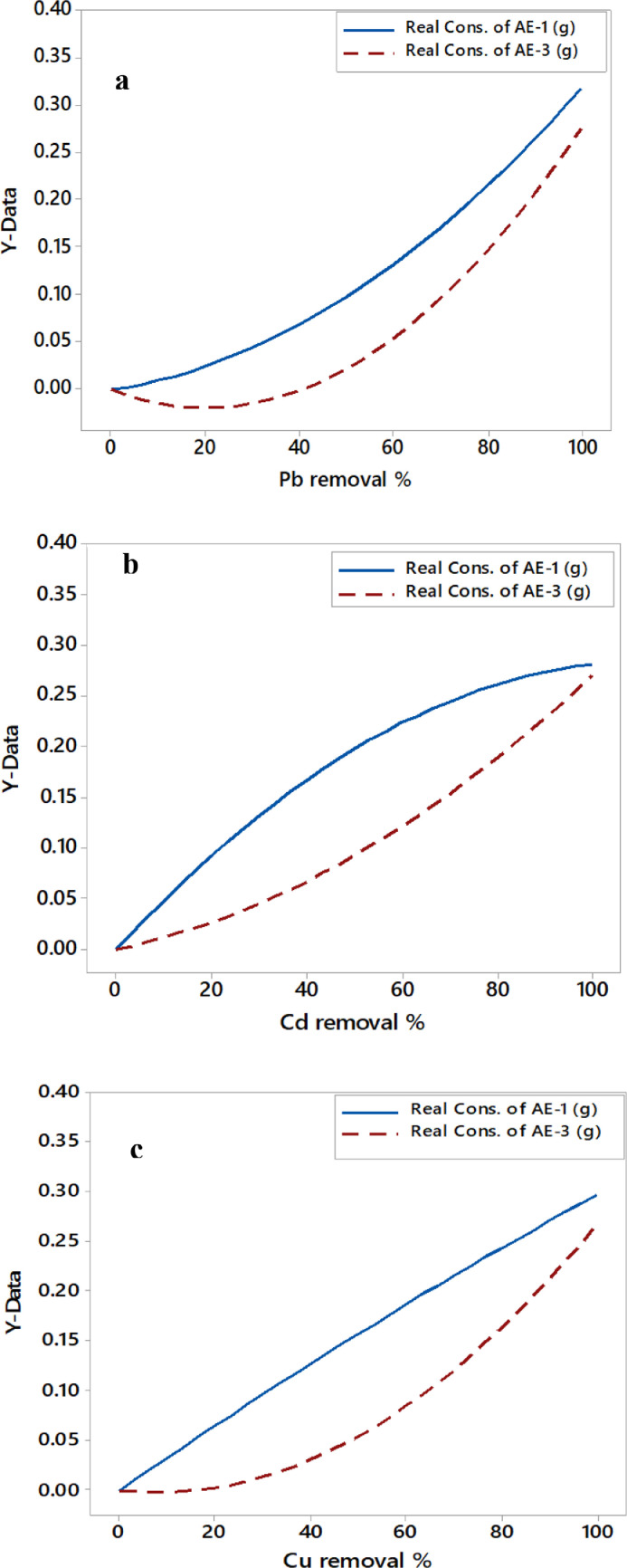
Removal efficiencies of toxic metals vs. the consumption of each anode.

As revealed in Table 6, the amount of electrode consumption was different for each anode because the AE-1 was directly connected to the electric source while the AE-3 was in the state of the bipolar electrode. Fig. 6 (a, b, and c) illustrates the consumed amount of each anode required to attain the removal efficiencies of toxic metals.

**Fig. 7:**
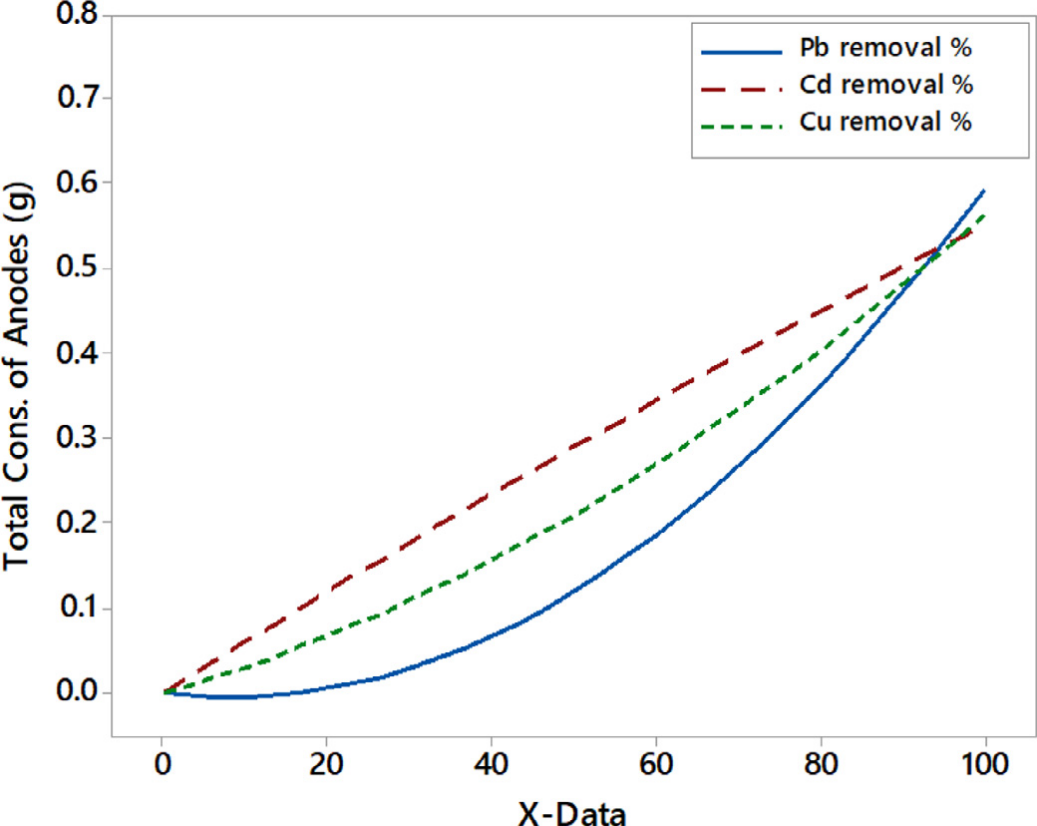
Removal efficiencies of toxic metals vs. the total consumption of anodes.

As observed in Fig. 6, the consumption of the AE-3 was similar in behavior for the removal of metals. The consumption of AE-3 was slower at the low value of removal efficiency, but then it was consumed rapidly until the highest remediation of metals was attained. Each metal removal had a different behavior of the AE-1 consumption. The Pb- removal% consumed a larger amount of AE-1 if compared to others, especially at the higher value of removal efficiency. However, the highest elimination of Cd metal depended on consuming AE-3 more than the consumption of the AE-1 electrode. The irregular behavior presented in Fig. 6 maybe refers to the formation issue of an oxide layer at some locations of electrodes. This layer occurred when the NaCl electrolyte could not remove this passivation because of the huge amount of tiny gas bubbles formed on the surface of the electrodes. As shown in Fig. 7, the lowest removal efficiency of Pb metal had the lowest total consumption of both anodes, but the highest Pb-removal efficiency was inverse. However, the highest removal efficiency of Cd metal has the highest total consumption of anodes. But the situation for Cu metal was in between. Interpreting these behaviors may refer to as the degree of the attractive force that occurred between the pollutants and the electro-coagulants formed [36,[58], [59], [60].

### Kinetic study

The order of reaction that occurs throughout any electrochemical reactor should be investigated. The kinetic modeling for the present configuration of electrodes is provided to obtain the rate constants of the EC process. First and second-order equations are presented in Eqs. (20) and (21) as follows:

(20) $$-ln\frac{C_{t}}{C_{i}}=k_{1}t$$

(21) $$\frac{1}{C_{t}}-\frac{1}{C_{i}}=k_{2}t$$

where C_i_ and C_t_ are the concentration of each metal at initial and time t, respectively, k_1_ and k_2_ are the rate constant for first order (min^−1^) and second order (m^3^. mol^−1^. min^−1^), respectively, and t is the reaction time in minutes.

The results presented in Table 12 revealed that Pb, Cd, and Cu metals removal obeyed the kinetic of first-order reaction in their behaviors based on the regression coefficient (R^2^). These results gave an additional advantage for the present EC reactor because the kinetic of second-order reaction is more slower and complicated compared to the first-order reaction.

**Table 12:** Summary of the present kinetic study.

Heavy Metals	Reaction order	Regression equations	k (mol/m^3^)^1-n^	R^2^
Pb	1	*y* = 0.1731 *x* + 0.8536	0.1731	**0.864**
	2	*y* = 4.9309 x - 111.63	4.9309	0.611
Cd	1	*y* = 0.1010 *x* + 0.0063	0.1010	**0.964**
	2	*y* = 0.1951 x - 1.1432	0.1951	0.776
Cu	1	*y* = 0.3021 *x* + 0.5989	0.3021	**0.905**
	2	*y* = 6.4166 x – 20.00	6.4166	0.635

### Thermodynamic parameters

The values of thermodynamic parameters have been estimated based on the temperature variation measured periodically throughout the EC reactor under the optimal conditions. Eq. (22) lists the estimated relation between the equilibrium constant (K_d_) and the reciprocal of the solution temperature.

(22) $$\log\;K_{d}=-5443.6\;\left(1/T\right)+24.905$$

The value of **Δ**H is estimated from the slope of Eq. (22) and other thermodynamics parameters are obtained from the following equations Eqs. (23) and ((24)) as follows:

(23) $$\Delta G=-\text{RT}\;\ln K_{d}$$

(24) ΔG=ΔH−TΔS

**Fig. 8:**
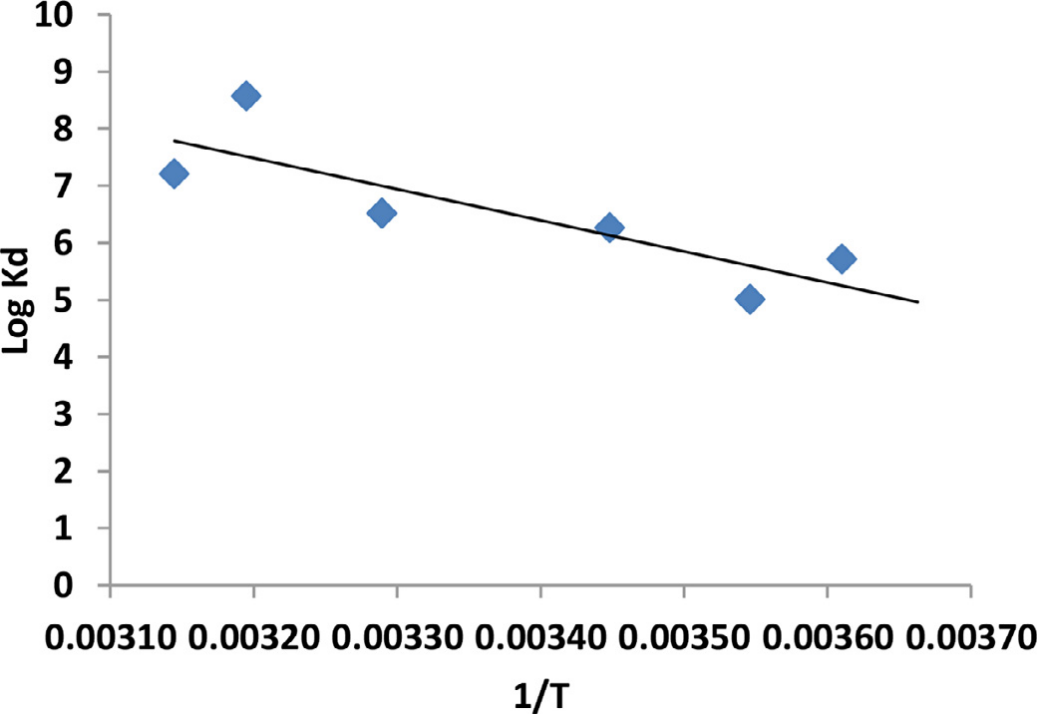
The logarithm of equilibrium constant vs. (1/T).

Based on Fig. 8, the value of **Δ**H is obtained from the slope of this line which equals 104.229 kJ/mol. The values of **Δ**G was negative, and **Δ**S was positive. The present EC process is endothermic, spontaneous nature, and random of irregularity at the liquid- solid interaction.

### Optimization with RSM-BBD

**Fig. 9:**
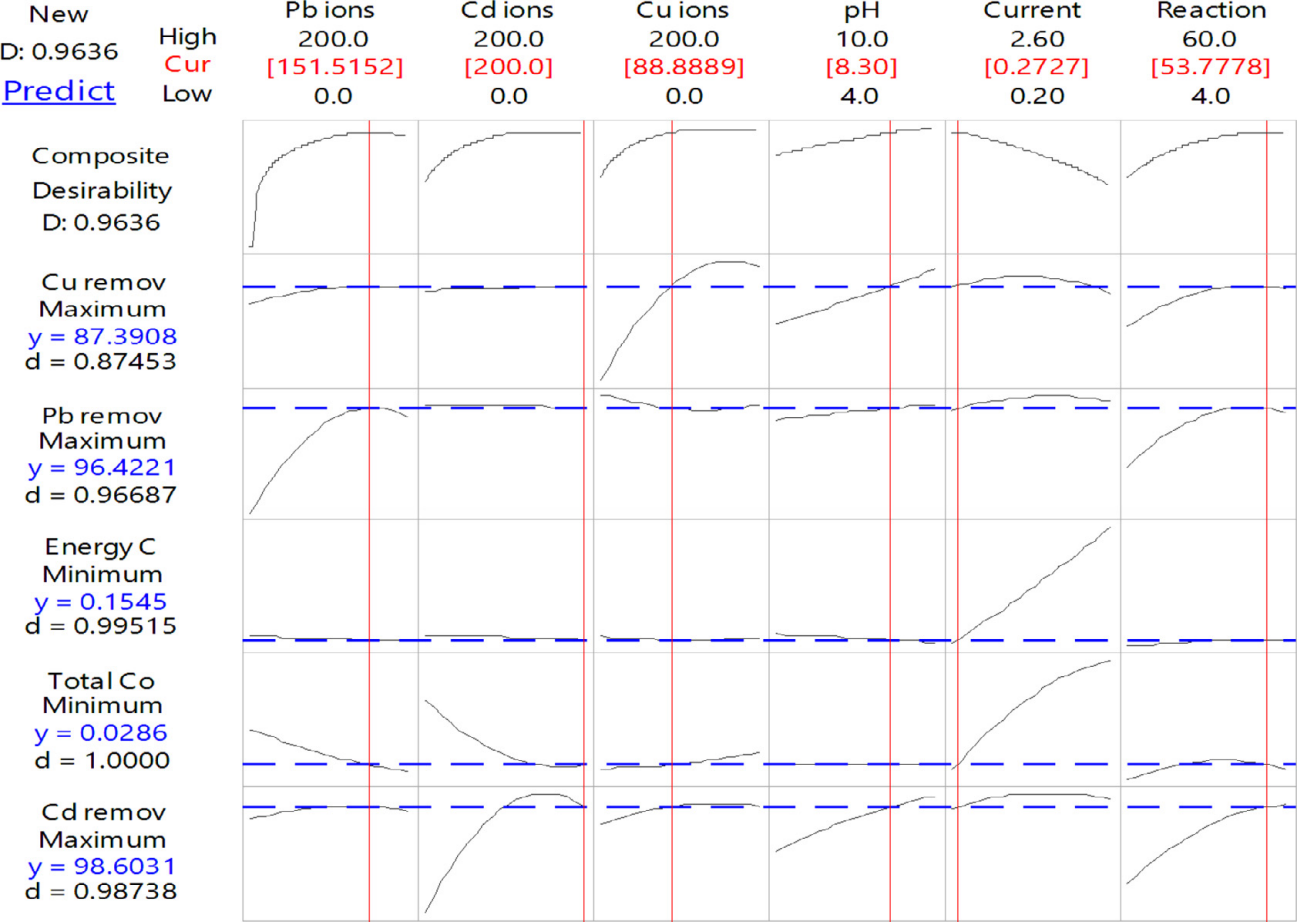
The optimization of the studied variables.

RSM-BBD has estimated the optimum conditions for the studied operating variables and the required responses using the Minitab-statistical software program. The optimization of Pb, Cd, and Cu-ions were chosen within the ranges, and it maximized the studied responses of removal efficiencies. However, the optimization of energy consumption and total consumption of electrodes was minimized. Fig. 9 and Table 13 show the optimization process of the studied variables and responses by considering all the operating variables.

**Table 13:** The values of the optimal studied variables and the required responses.

Variables and responses	Values	Composite Desirability (D-value)
Pb Concentration (ppm)	151.52	0.9636
Cd Concentration	200	0.9636
Cu Concentration	88.889	0.9636
pH	8.30	0.9636
Current (A)	0.27	0.9636
Reaction time (min)	53.78	0.9636
Pb removal%	96.42	0.9667
Cd removal%	98.60	0.9874
Cu removal%	87.39	0.8745
Real consumption of electrodes (g)	0.029	1.0000
Energy consumption (kWh/m^3^)	0.155	0.9952

**Fig. 10:**
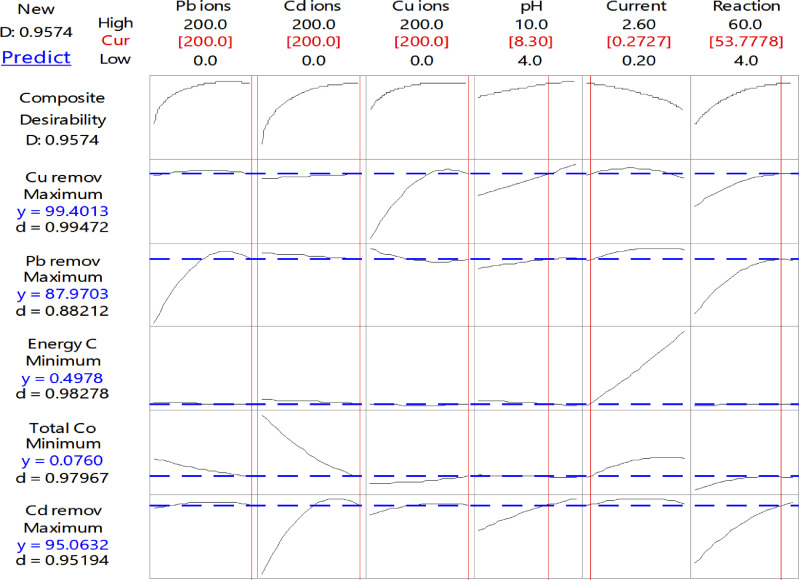
The optimization of the studied variables for total amounts of heavy metals.

Since the target of any treatment process is to remove the initial concentration of all pollutants found in the wastewater, Fig. 10 and Table 14 provide the observed values of the studied responses when all concentrations of heavy metals are proposed to be the optimal values.

**Table 14:** The values of the optimal studied variables and the required responses.

Variables and responses	Values	Composite Desirability (D-value)
Pb Concentration (ppm)	200	0.9574
Cd Concentration	200	0.9574
Cu Concentration	200	0.9574
pH	8.30	0.9574
Current (A)	0.27	0.9574
Reaction time (min)	53.78	0.9574
Pb removal%	87.97	0.8821
Cd removal%	95.06	0.9519
Cu removal%	99.40	0.9947
Real consumption of electrodes (g)	0.076	0.9796
Energy consumption (kWh/m^3^)	0.498	0.9828

Table 15 summarizes some previous works that concerned the removal of heavy metals from wastewater using different configurations of electrodes. This table lists the type of metal used for electrodes regardless of the configuration of them, the optimal values of the operating variables, and the highest removal achieved.

**Table 15:** Summary of some previous studies used EC for heavy metals removal from wastewater.

References	Pollutants	Metal of electrodes (Anode/Cathode)	Optimum conditions	Removal Efficiency%
[5]	Pb	Al/Al	33 A/m^2^, pH 7, 30 min	96%
[6]	Cu, Ni, Pb	Al/Fe	0.026 A/cm2, pH 6.32, HRT: 60 s,	95%
[7]	Cr (VI)	Al/Fe	14 mA/cm^2^, 90 min, pH 7	92%
[8]	Ni	Al/Cu	5 V, 76.5 min	>90%
[61]	Cu, Zn, Ni, Cr	Al/Fe	40 mA/cm^2^, 60 min	Cu, Zn, Ni: 97%; Cr: >80%
[62]	Cu, Pb, Cd	Ag/Pt	pH 7, 6.3 ml/min	80%
[63]	Cd, Cu, Ni	Al\Fe	30 mA/cm^2^, 90 min, pH 7	>98%
[64]	Cu, Ni, Zn, Mn	Fe/Fe	25 mA/cm^2^, 25 min	96%
Present study	Pb, Cd, and Cu	Al/Al	0.75 mA/cm^2^, 54 min, pH 8.3	>99

### Cost estimation

The estimation of the operating cost is remarkably essential to assess the performance of the present design of the EC reactor used to remove toxic heavy metals from wastewater. The main issue of this subject is the estimation of electrodes and electrical energy consumption that should be taken into consideration. Eq. (25) is used to estimate the cost of the present consumption of electrodes and energy.

(25) $$\text{Total}\;\text{cost}=A_{1}\times \text{ENC}\;\left[\text{kWh}/m^{3}\right]+A_{2}\times \text{Real}\;\text{consumption}\;\text{of}\;\text{electrodes}\;\left[g/m^{3}\right]$$

where A_1_ and A_2_ are the prices of unit electrical energy [$/kWh] and unit weight of aluminum [$/g], respectively. At the research time, they equal 0.013 $/kWh and 2.74 × 10^–3^ $/g, respectively, according to the local price.

Since the real consumption of electrodes under the optimal conditions was 0.55 g and the energy consumption was 12.71 kWh/m^3^, the total cost was 0.167 $ per each cubic meter of the treated wastewater.

## Conclusions

Industrial activities are discharging huge amounts of metal wastewater into the environment without efficient treatment. This study employed an electrocoagulation reactor to eliminate multi-toxic metals from synthetic wastewater using four concentric cubic electrodes made of aluminum. The anodes have a perforated shape, while the cathodes do not. This work has studied several operating variables using the RSM-BBD design method to evaluate the responses of pollutants removal efficiencies and the consumption amounts of energy and electrodes. The highest removal efficiencies of Pb, Cd, and Cu metals were 99.73%, 98.54%, and 98.92%, respectively, with an energy consumption of 12.71 kWh/m^3^ and electrodes consumption of 0.55 g at pH 10, applied current of 1.4 A, and reaction time of 60 min with a significantly low cost. All reactions of metal removal that occurred throughout this reactor obey the kinetic of a first-order reaction. Thermodynamics parameters of present electrocoagulation removal of heavy metals indicate an endothermic, spontaneous nature, and random irregularity at the liquid-solid interaction. The present design of the EC reactor proved the ability of the electrocoagulation process to eliminate heavy metals from wastewater with low amounts of energy and electrode consumption.

## Declaration of Competing Interest

The authors declare that they have no known competing financial interests or personal relationships that could have appeared to influence the work reported in this paper.

## Data Availability

The data that has been used is confidential.
